# Monte Carlo simulation study of an *in vivo* four-dimensional tracking system with a diverging collimator for monitoring radiation source (Ir-192) location during brachytherapy: proof of concept and feasibility

**DOI:** 10.3389/fphys.2024.1302301

**Published:** 2024-03-25

**Authors:** Geon Oh, Jeongshim Lee, Hunjung Kim, Woochul Kim, Sangwon Kang, Jinbeom Chung, Seonghoon Jeong, Hakjae Lee, Myonggeun Yoon, Boram Lee

**Affiliations:** ^1^ Department of Bioengineering, College of Health Sciences, Korea University, Seoul, Republic of Korea; ^2^ Department of Radiation Oncology, Inha University Hospital, Incheon, Republic of Korea; ^3^ Department of Radiation Oncology, Seoul National University Bundang Hospital, Seongnam, Gyeonggi, Republic of Korea; ^4^ Department of Neurology, Inje University Ilsan Paik Hospital, Goyang, Gyeonggi, Republic of Korea; ^5^ ARALE Laboratory Co., Ltd., Seoul, Republic of Korea

**Keywords:** HDR brachytherapy, Ir-192 source, *in vivo* 4D tracking system, three-dimensional positioning, Monte Carlo (MC)

## Abstract

**Introduction:** The aim of this study was to demonstrate the potential of an *in vivo* four-dimensional (4D) tracking system to accurately localize the radiation source, Iridium-192 (Ir-192) in high-dose rate brachytherapy.

**Methods:** To achieve time-dependent 3D positioning of the Ir-192 source, we devised a 4D tracking system employing multiple compact detectors. During the system’s design phase, we conducted comprehensive optimization and analytical evaluations of the diverging collimator employed for detection purposes. Subsequently, we executed 3D reconstruction and positioning procedures based on the 2D images obtained by six detectors, each equipped with an optimized diverging collimator. All simulations for designing and evaluating the 4D tracking system were performed using the open-source GATE (v9.1) Monte Carlo platform based on the GEANT4 (v10.7) toolkit. In addition, to evaluate the accuracy of the proposed 4D tracking system, we conducted simulations and 3D positioning using a solid phantom and patient data. Finally, the error between the reconstructed position coordinates determined by the tracking system and the original coordinates of the Ir-192 radiation source was analyzed.

**Results:** The parameters for the optimized diverging collimator were a septal thickness of 0.3 mm and a collimator height of 30 mm. A tracking system comprising 6 compact detectors was designed and implemented utilizing this collimator. Analysis of the accuracy of the proposed Ir-192 source tracking system found that the average of the absolute values of the error between the 3D reconstructed and original positions for the simulation with the solid phantom were 0.440 mm for the x coordinate, 0.423 mm for the y coordinate, and 0.764 mm for the z coordinate, and the average Euclidean distance was 1.146 mm. Finally, in a simulation based on data from a patient who underwent brachytherapy, the average Euclidean distance between the original and reconstructed source position was 0.586 mm.

**Discussion:** These results indicated that the newly designed *in vivo* 4D tracking system for monitoring the Ir-192 source during brachytherapy could determine the 3D position of the radiation source in real time during treatment. We conclude that the proposed positioning system has the potential to make brachytherapy more accurate and reliable.

## 1 Introduction

High-dose rate (HDR) brachytherapy using Iridium-192 (Ir-192) as the radiation source is widely employed in the treatment of diverse cancers (Suntharalingam et al., 2005; Venselaar et al., 2012). In this technique, a remote loading device is used to precisely place a highly active Ir-192 source within an applicator in the targeted treatment region. HDR brachytherapy facilitates the delivery of concentrated radiation doses to the intended target volume, thereby minimizing radiation of healthy tissues due to the sharp decrease of dose with increasing distance (Challapalli et al., 2012; Morton and Hoskin, 2013).

Although HDR brachytherapy has advantages, including exceptional dose concentration, it also has drawbacks. Of particular concern are medical accidents arising from mispositioning of the radiation source, some leading to patient fatalities (Protection, 2001; Thomadsen et al., 2003; Ashton et al., 2005; Valentin, 2005; Richardson, 2012). To prevent such accidents and to optimize the potential of HDR brachytherapy, the ability to precisely monitor the position of the Ir-192 source is critical (Majewski et al., 1999; Nakano et al., 2005).

To date, several methods, including X-ray imaging systems (Nakano et al., 2005; Nose et al., 2017), scintillation cameras (Majewski et al., 1999; Duan et al., 2001; Batič et al., 2010; Safavi-Naeini et al., 2015), non-imaging systems (Kertzscher et al., 2014), and gamma cameras (Yamamoto et al., 2019; Nagata et al., 2021), have been proposed for accurately monitoring the position of the radiation source within the patient. These methods enable the identification of the source’s movement and position on a 2-dimensional image but cannot accurately determine the position in 3 dimensions at the given time through a single detection. This uncertainty in 3D positioning not only makes it difficult to guarantee the accuracy of HDR brachytherapy but can also lead to unexpected problems. As a solution, a 4-dimensional (4D) tracking system has been proposed that determines the motion and position of the Ir-192 source in 3D through simultaneous detection at multiple detectors, complementing existing treatment verification methodologies.

Whereas the 3D system considers only the three Cartesian spatial coordinates, the 4D system includes the additional dimension of time (Li et al., 2008; Reader and Verhaeghe, 2014). For a radiation source tracking system based on a 4D framework to be of practical utility in clinical practice, the system must be able to simultaneously acquire sufficient data to determine the location of the radiation source within the patient’s body in 3D, through a single detection event. This enables the derivation of 3D coordinates for each time step.

Furthermore, when configuring these 4D systems, patient safety and convenience must be foremost considerations. The process of acquiring 3D images must inherently preclude unintentional accidents. Especially when employing a detection system comprised of multiple detectors for tracking the radiation source, the system’s design must safeguard against potential mishaps. Therefore, the crafting of a compact system assumes paramount significance, forming a crucial line of defense against any inadvertent accidents during the radiation source tracking process.

Here, we propose a new *in vivo* 4D tracking system for HDR brachytherapy. By integrating 3D reconstruction techniques based on 2D images from multiple compact detectors positioned at various angles, our aim was to obtain 3D motion and positioning information about the Ir-192 source through a single, simultaneous detection event. Additionally, to design such a compact detector system, we optimized the performance of the detection systems using a compact diverging collimator, a type of collimator mainly used when the object to be detected is larger than the detected image, for acquiring improved 3D images. Finally, the proposed 4D tracking system was evaluated by comparing the coordinates derived using the tracking system with the actual position coordinates of an Ir-192 source.

## 2 Methods

### 2.1 Optimization of the diverging collimator for a compact detector system

**FIGURE 1:**
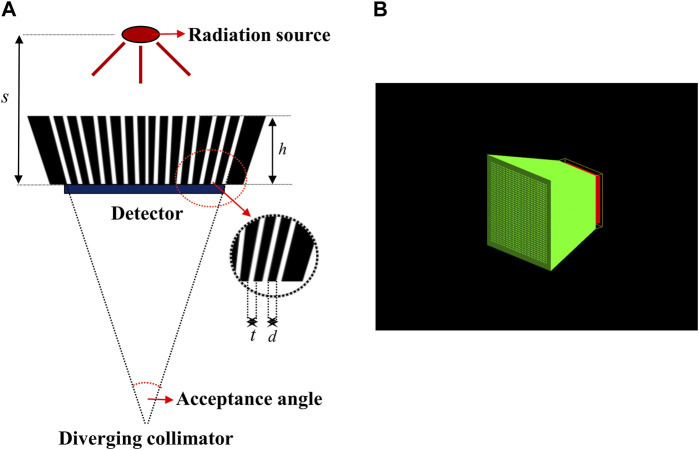
**(A)** Parameter of diverging collimators **(B)** Geometry of diverging collimator in geant4 application for tomographic emission (GATE).

To design the diverging collimator for use in detecting an Ir-192 radiation source, we employed Monte Carlo simulations using GATE v9.1, a widely recognized gamma imaging simulation tool (Jan et al., 2004). First, the optimization process used diverging collimators made of pure tungsten (atomic number 74, Atomic weight 183.8 u, and density 19.3 g/cm3) with a 38 × 38 hole array structure. For the detector system, the overall dimensions of the scintillator based on lutetium yttrium oxyorthosilicate (LYSO) was set to 25.8 mm × 25.8 mm x 4 mm, and the simulation was based on a 38 × 38 pixel array with a resolution of 0.5 mm × 0.5 mm x 4 mm. Additionally, throughout all simulations, a cylindrical Ir-192 source with a diameter of 0.6 mm and a height of 3.5 mm was positioned at a distance of 500.0 mm from the detector surface. The activity of the Ir-192 source was configured to be 360 mCi. Next, for the optimization of the diverging collimator, the height (h) of the collimator was varied from 20 to 50 mm in 5 mm increments, while the sum of the hole size (t) and septal thickness (d) between the holes was kept constant at 0.6 mm, and the thickness of the septal (t) was varied from 0.1 to 0.4 mm. Figure 1 shows each parameter of the diverging collimator and the configuration of the collimator in the Monte Carlo simulations. Furthermore, to enhance spatial resolution and achieve images with reduced scatter fraction, we set the center energy value of the energy window at 300 keV, accompanied by an energy window size of 50 (Nagata et al., 2022). These settings were informed by previous findings on the energy range optimization for gamma camera systems employing an Ir-192 source (Nagata et al., 2022).

**FIGURE 2:**
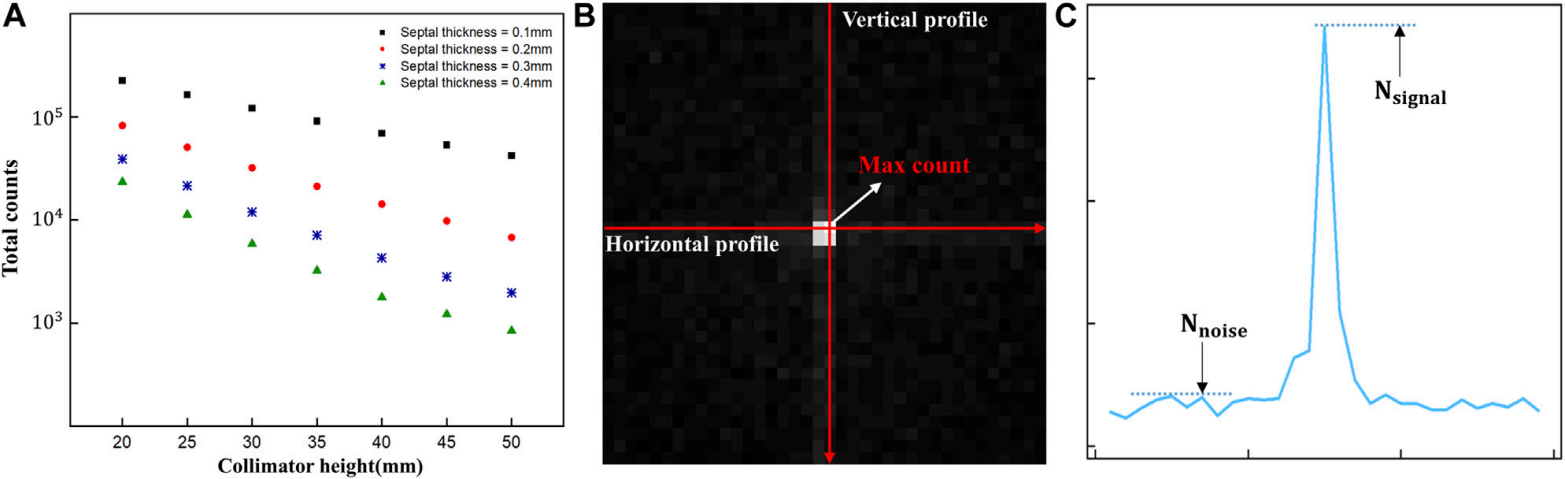
**(A)** Total counts of diverging collimators **(B)** Acquired 2D gamma image and profile direction **(C)** Parameters of the signal-to-noise measurements in gamma images.

The 2D gamma images acquired using the detector were then assessed using the full-width at half-maximum (FWHM) method and the signal-to-noise ratio (SNR) to determine the spatial resolution and image quality. Before evaluating the acquired images through simulation, we first checked the total counts of the acquired images based on previous research showing that detection of Ir-192 sources with scintillation cameras or gamma cameras is hampered by problems such as difficulty in collimation due to strong radioactivity and saturation of the electronics circuit (Figure 2A) (Nagata et al., 2020). In addition, to evaluate the acquired images, vertical and horizontal profiles were made based on the maximum count, i.e., the pixel with the highest value in the image (Figure 2B), and the SNR was calculated using the following equation (Han et al., 2022):

$$\text{SNR}=\left(N_{\text{signal}}‐N_{\text{noise}}\right)\;/\;\sqrt{N_{\text{noise}}}\cdots$$ (1)

where $N_{\text{signal}}$ is the count of the signal at the profile peak and $N_{\text{noise}}$ is the count of the noise in the background (Figure 2C). Finally, we employed an analysis tool based on Python code to accurately calculate the FWHM and SNR.

### 2.2 Performance evaluation of the optimized diverging collimator

The spatial resolution and image quality of a diverging collimator tend to deteriorate as the position of the radiation source being detected deviates from the center of the field of view (FOV) (Cha et al., 2021). Consequently, we conducted additional assessments of the optimized diverging collimator to ascertain its performance across varying source positions (Cha et al., 2021). Table 1 presents the geometric parameters of the optimized diverging collimator. To assess its performance, we moved the source while maintaining the remaining conditions consistent with those identified during the optimization procedure. Specifically, we displaced the source in 1-cm intervals along the positive *y*-axis and diagonally in 1-cm intervals along both the positive *z*-axis and positive *y*-axis directions, resulting in a cumulative shift of 8 cm for each type of displacement. The 2D gamma images obtained at the various source positions were assessed using the same FWHM and SNR as those employed during the optimization process.

**TABLE 1:** Geometric parameters of the optimized diverging collimator.

Parameter	Value
Distance from the collimator surface to the source [cm]	50.0
Hole size (d) [mm]	0.3
Septal thickness (t) [mm]	0.3
Height of the collimator [mm]	30
Acceptance angle [°]	45
Hole array	38 × 38

### 2.3 Design of the 4D tracking system with multiple compact detectors

**FIGURE 3:**
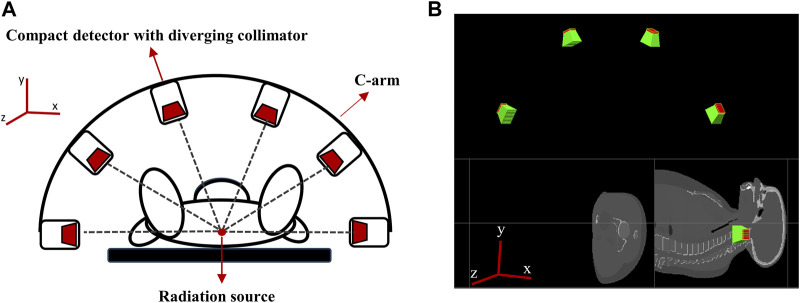
**(A)** Schematic of the proposed 4D tracking system, **(B)** Geometry of multiple compact detector system in GATE.

To acquire 3D data on the position of the Ir-192 source in a single detection event, we needed to acquire two or more 2D images captured from different angles. For this reason, a multiple detector system capable of simultaneous detection from multiple angles was devised. The system’s hardware was designed based on two main considerations. First, miniaturization of the detectors was required to enhance patient safety and clinical workflow. Second, the resolution of the 4D tracking system needed to be sufficient for clinical use. To enhance clinical applicability, we designed a multi-detector configuration consisting of 6 compact detectors that were size small enough to allow attachment to the X-ray imager (e.g., C-arm) currently used for brachytherapy. Figure 3 shows a schematic of the proposed 4D tracking system and the geometry used for the simulation in GATE. The intrinsic resolution of each detector was configured with an independent pixel size of 0.5 mm by employing a 0.1 mm specular reflector, with an interpixel distance of 0.6 mm. Although this structure is quite compact, the resolution deteriorates as the collimator approaches the detecting object due to the characteristics of a diverging collimator. When the detector is expanded to the center of the object, the detectable area of the detector increases from its actual size of 0.6 mm to a maximum of 6 mm. However, when the matrix size is the same, an increase in the detection area ultimately leads to a decrease in spatial resolution. Multiple detectors arranged at various angles can enhance the x- and *y*-axis resolutions. The *z*-axis resolution was improved through cross-positioning of the detectors in the *z*-axis direction.

In more detail, the tracking system consists of six detectors that are equally spaced along a semicircular structure that surrounds the target in a co-planar configuration (see Figure 3A). The detectors are positioned relative to the treatment room coordinate system, assuming an axial plane in supine patient. Specifically, two detectors are placed at a distance of 50 cm along the positive and negative *x*-axes from the origin (0,0,0) and the other four detectors are positioned at intervals of 36°, resulting in detectors at 270, 306, 342, 18, 54, and 90° in the xy plane (see Figure 3A).

**FIGURE 4:**
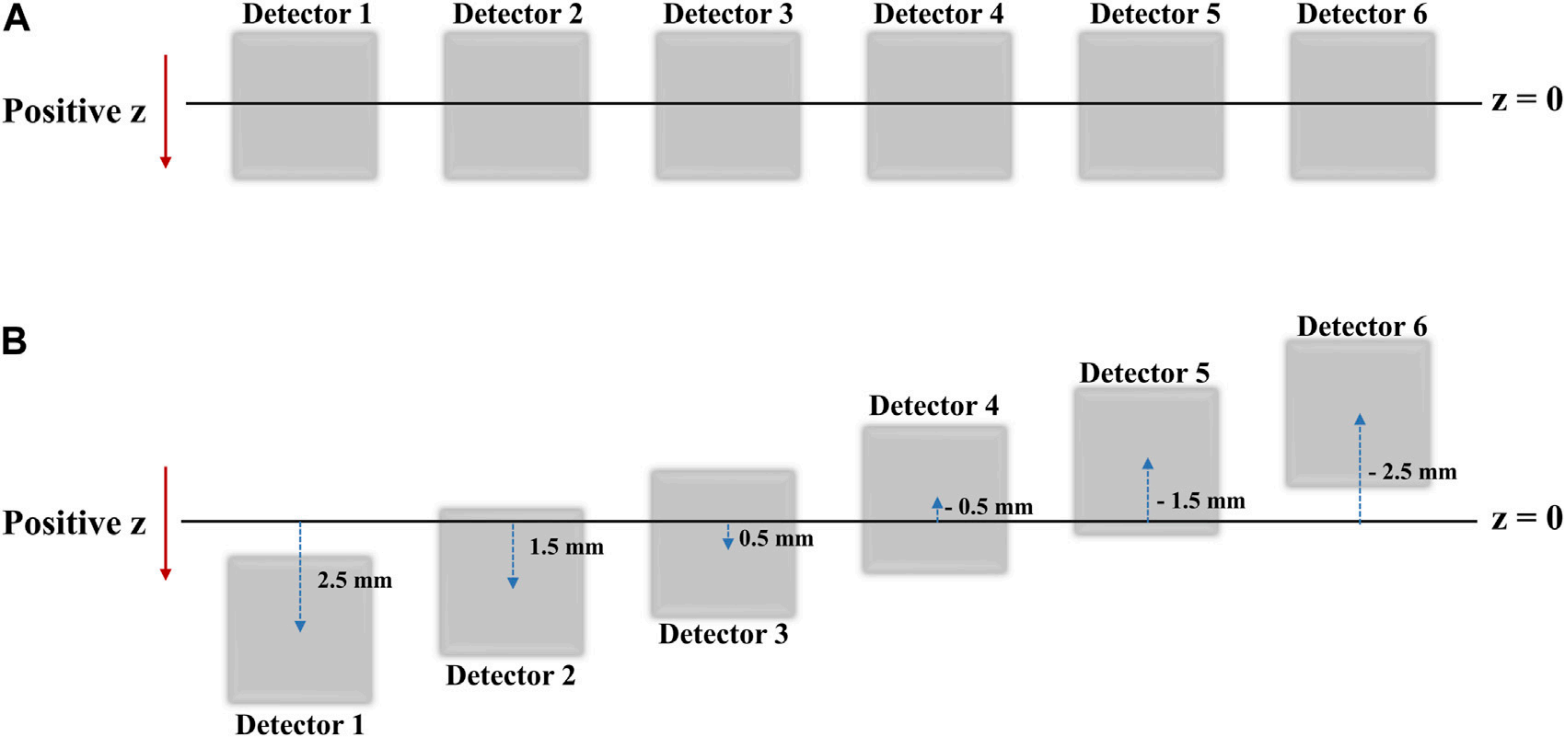
**(A)** Detector positions as seen from the xy-plane view **(B)** Detector positions as seen from the XY plane view after shifts in the *Z* direction for high resolution 2D image acquisition.

In practice, when the six detectors are placed in the same xy plane (i.e., at the same z-coordinate), the intrinsic resolution in the *z*-axis direction of the radiation source is the same for each detector, there is a limit to the accuracy with which the coordinates of the radiation source can be determined. Therefore, to more precisely determine the radiation source position in the *z* direction, we rearranged the six detectors to have different *z*-axis coordinates. The data recorded by the repositioned detectors (Figure 4) were processed using a source location tracking algorithm to enhance the resolution.

### 2.4 4D tracking algorithm for positioning of the Ir-192 source

To enhance the resolution of the 2D gamma images acquired by the tracking system, we utilized a property of Gaussian filtering known as the up-sampling effect. Gaussian filtering adjusts each pixel value smoothly by considering the surrounding pixel values, thereby emphasizing high-resolution features in the image. Through this approach, we were able to capture crucial details that might be missed in low-resolution images and to minimize positional errors, ultimately achieving more accurate results. Based on these Gaussian filtered images, reconstruction and source positioning were performed.

Next, we utilized the ASTRA Toolbox for Python to generate 3D tomographic objects for each simulation case. The 4D tracking algorithm was developed to enable GPU calculation using CUDA to reduce computation time. The tracking algorithm also supported multiple reconstruction algorithms, including filtered back projection (FBP) and maximum likelihood expectation maximization (MLEM) (Van Aarle et al., 2016).

For the 3D reconstruction, we employed an NVIDIA GeForce RTX 3090Ti GPU with 24 GB of memory and utilized the SIRT3D_CUDA algorithm, one of the MLEM methods available in the ASTRA Toolbox. The CUDA-based Simultaneous Iterative Reconstruction Technique (SIRT) method dramatically reduces the time required compared to the basic SIRT method while still showing excellent accuracy. We also performed the reconstruction with different numbers of iterations and found that the results were the same when the iteration number was 3 or higher. For this reason, we set the iteration number to 3. The method is formally represented by the equation (Gilbert, 1972; Andersson, 2016):

$${\text{X}}^{\text{k}+1}={\text{X}}^{\text{k}}+CA^{T}R\left(b‐A{\text{X}}^{\text{k}}\right)\cdots$$ (2)

where R and C are diagonal matrices with scaling values. R can be a diagonal matrix with correction values for the distance the ray passes through the reconstruction volume, which is the row sums of the weight matrix, r_ii_ = 1/ΣM_j_₌₁ w_ij_. C can also be a diagonal matrix but with the column sums of the weight matrix, c_jj_ = 1/ΣN_i_₌₁ w_ij_. The transposed matrix, A^T^, back projects the projection images onto the reconstruction area. The term (b − AX^k^) is the error between b (the projections) and AX^k^ (the current reconstruction, X^k^, is forward projected).

To determine the 3D position of the Ir-192 source, we calculated the center of mass of the reconstructed object volume. The equation for calculating the centroid (center of mass) of a set of n voxels in three-dimensional space with positions (x_i_, y_i_, z_i_) and weights (w_i_) is as follows:

$$\left(x,y,x\right)=\left(\Sigma \left(w_{i}\;*\;x_{i}\right)\;/\;\Sigma w_{i},\Sigma \left(w_{i}\;*\;y_{i}\right)\;/\;\Sigma w_{i},\Sigma \left(w_{i}\;*\;z_{i}\right)\;/\;\Sigma w_{i}\right)\cdots$$ (3)

Using this equation, we derived the reconstructed position coordinates of the Ir-192 source in 3D space. The accuracies of the x, y and z coordinate values were evaluated as the absolute value of the error between the actual and reconstructed positions of the Ir-192 source in the x, y, and z directions, respectively. We also measured the Euclidean distance between the actual and reconstructed position vectors of the Ir-192 source (Dokmanic et al., 2015).

### 2.5 Evaluation of the tracking system accuracy

Initially, we assessed the performance of the tracking system in simulations. The primary objective was to ascertain the system’s accuracy within 3D coordinate space by changing the position of the Ir-192 radiation source along the x, y, and *z*-axes. The source’s position spanned from the center of the FOV to a distance of 8 cm in 1 cm intervals along each axis, while maintaining consistent conditions. To validate the practical viability of clinical implementation, we used data from patients who underwent tandem and ovoids brachytherapy to identify the original positions of a total of 19 Dwell positions of sources (9 source positions in the tandem applicator and 5 positions each in ovoids 1 and 2). Subsequently, we performed detection simulations and 3D positioning emulating real treatment scenarios with our tracking system. Finally, we quantitatively analyzed the discrepancies between the position coordinates derived from the reconstruction process and the initial coordinates of the Ir-192 radiation sources for all the simulated locations.

## 3 Results

### 3.1 Optimization of the diverging collimator for a compact detector system

**FIGURE 5:**
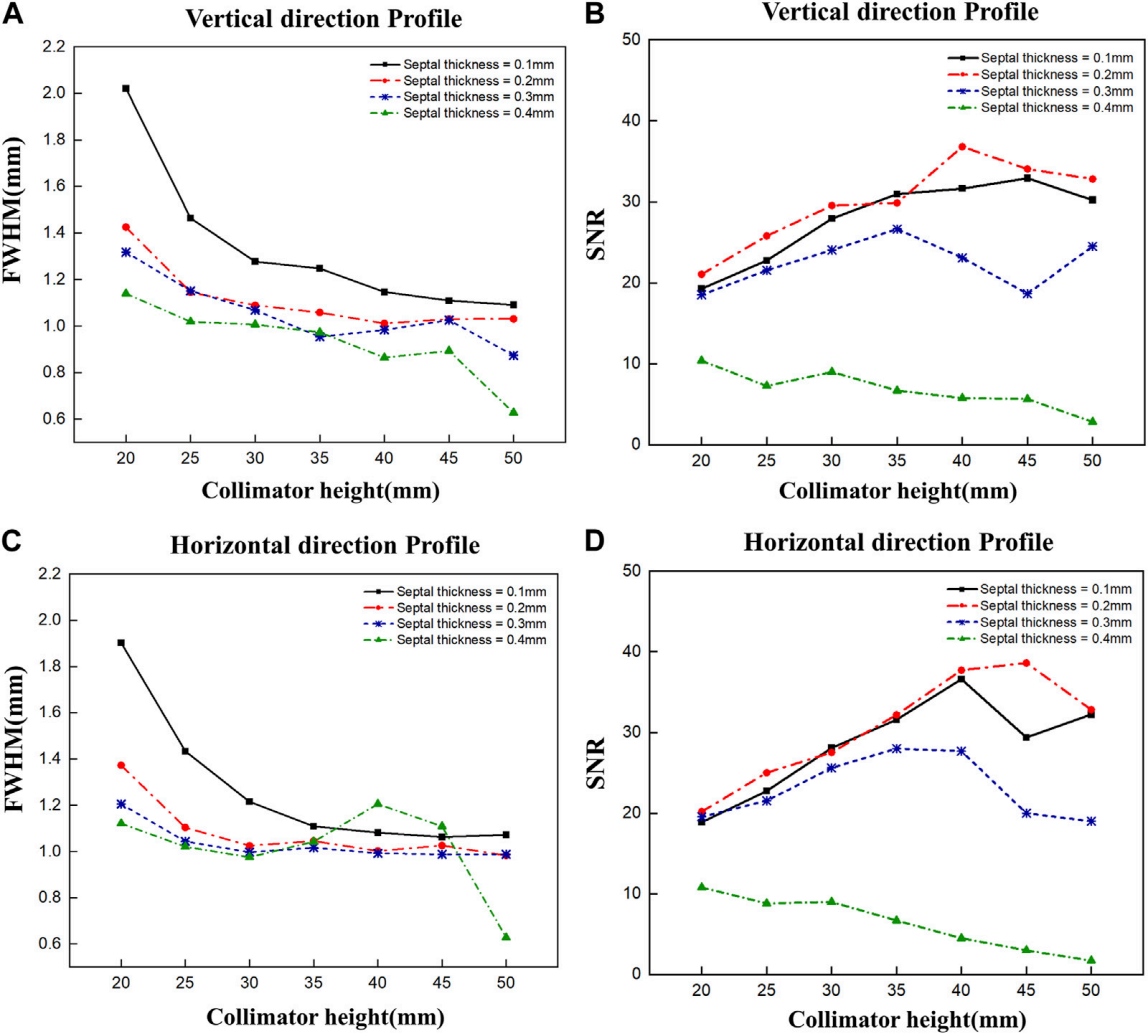
FWHM and SNR values of 2D gamma images obtained by varying the septal thickness and the height of the diverging collimator. FWHM **(A)** and SNR **(B)** when profiled vertically, and FWHM **(C)** and SNR **(D)** when profiled horizontally.

To optimize the diverging collimator, we determined the FWHM and SNR when profiled in the vertical and horizontal directions using collimators with varying heights and septal thicknesses (Figure 5). Overall, the vertical and horizontal profiles showed similar results in terms of FWHM and SNR. However, the SNR was very low at a septal thickness of 0.4 mm, resulting in poor image quality, and the FWHM was large at a septal thickness of 0.1 mm, resulting in poor spatial resolution. When we examine the trends as a function of collimator height, we find that the FWHM decreases with increasing collimator height for all septal thicknesses and converges to a constant value at a height of 30–35 mm, similar to the theoretical trend of resolution with diverging collimator height (Muehllehner, 1969). These results indicate that a septal thickness of 0.2 mm–0.3 mm and a collimator height of 30 mm–35 mm yield relatively favorable SNR outcomes coupled with low FWHM values. Given the robust radioactivity of the Ir-192 source, as well as the challenges associated with elevated total counts, we chose a septal thickness of 0.3 mm on account of its lower total count compared to the 0.2 mm option. Thus, to achieve a detector configuration prioritizing low weight and compactness, the optimal diverging collimator for detecting the Ir-192 source was found to have a septal thickness of 0.3 mm and a height of 30 mm (Table 1).

### 3.2 Performance of the optimized diverging collimator

**FIGURE 6:**
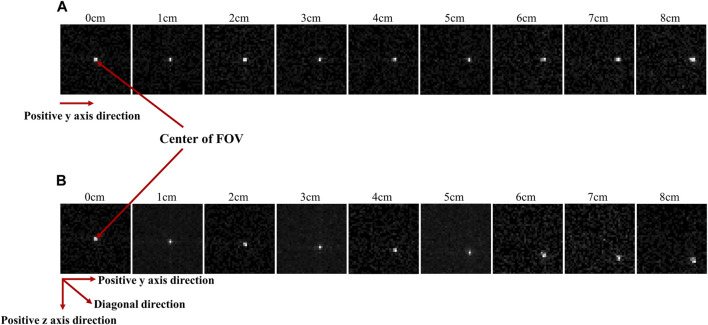
Gamma images acquired when the radiation source was repositioned to the positive *y* direction **(A)** and diagonal direction **(B)** from the center of the FOV.

Figure 6 presents images acquired using the optimized diverging collimator. These images were obtained by sequentially moving the position of the Ir-192 radiation source in both the positive *y*-axis (Figure 6A) and diagonal direction (Figure 6B). In the acquired images, a clearer distinction between source and background was observed for source displacements up to 6 cm; however, when the source was moved 7 cm and 8 cm, the source became blurred.

**FIGURE 7:**
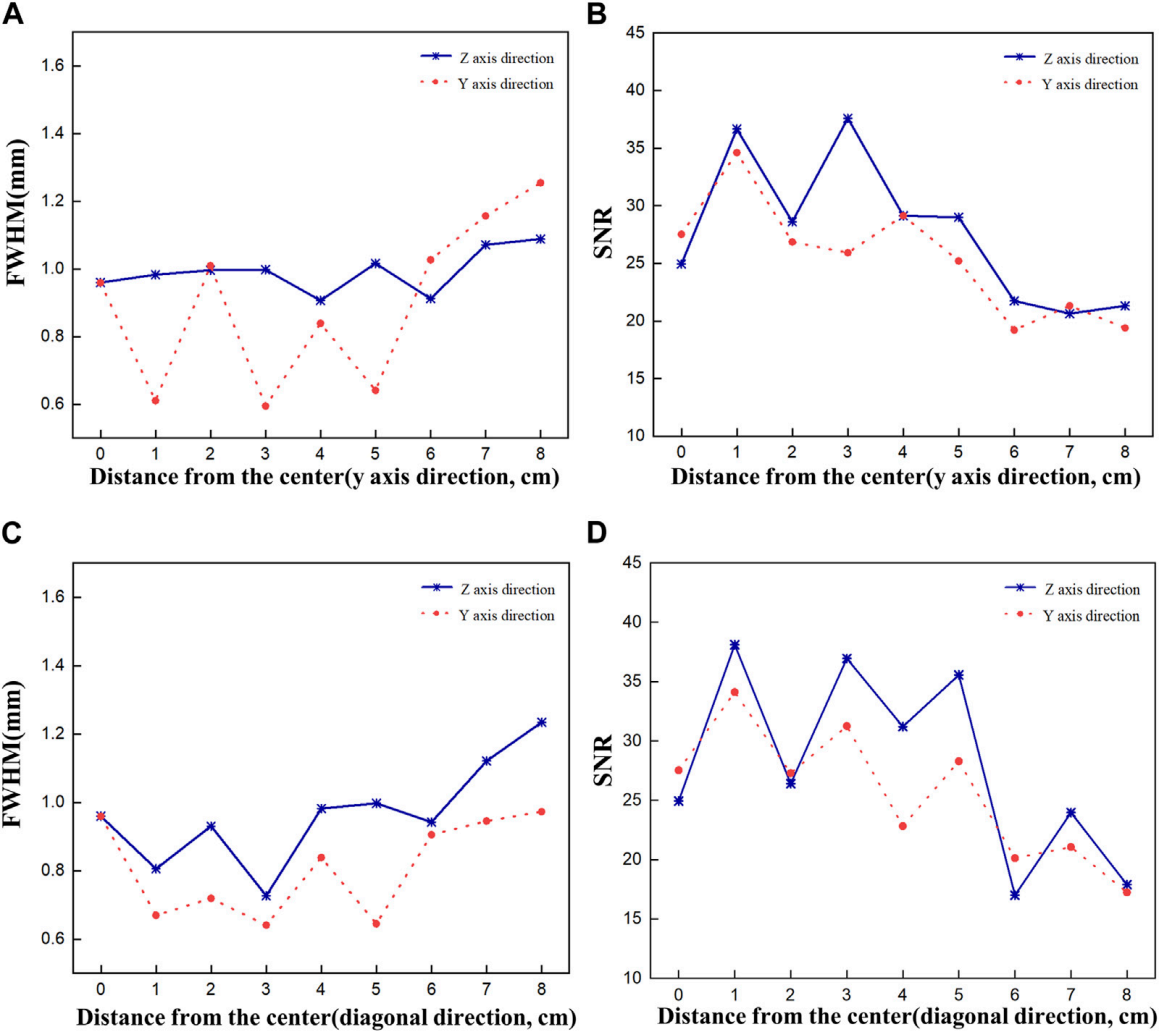
FWHM and SNR values of a 2d gamma image acquired while moving the source by 8 cm to evaluate the performance of the optimized collimator. FWHM **(A)**, SNR **(B)** when the source is moved in the y-axis direction, and FWHM **(C)**, SNR **(D)** when the source is moved in the diagonal direction.

Figure 7 shows plots of FWHM and SNR as a function of source displacement for the acquired 2D images. Figures 7A, B show the FWHM and SNR values when the Ir-192 source was moved in the positive *y*-axis direction, and Figures 7C, D show the FWHM and SNR values when the radiation source was moved in the diagonal direction. First, as an overarching characteristic, profiling along the *z*-axis direction resulted in higher FWHM values (indicative of lower spatial resolution) compared to profiling along the *y*-axis direction. Also, both directions exhibited similar trends with respect to SNR. For source displacements in both the *y*-axis and diagonal directions, the FWHM was consistently less than or near 1 mm across all examined source positions up to a displacement of 6 cm. Furthermore, the SNR values demonstrated robust performance except for the displacement of 6 cm. On the other hand, as the Ir-192 source displacement of increased from 6 to 8 cm, a linear increase in FWHM was observed along with low SNR values. This observation aligns with the inherent characteristics of the diverging collimator, whereby its performance diminishes as the radiation source to be detected moves further away from the center of the FOV.

### 3.3 Evaluation of tracking system accuracy

**FIGURE 8:**
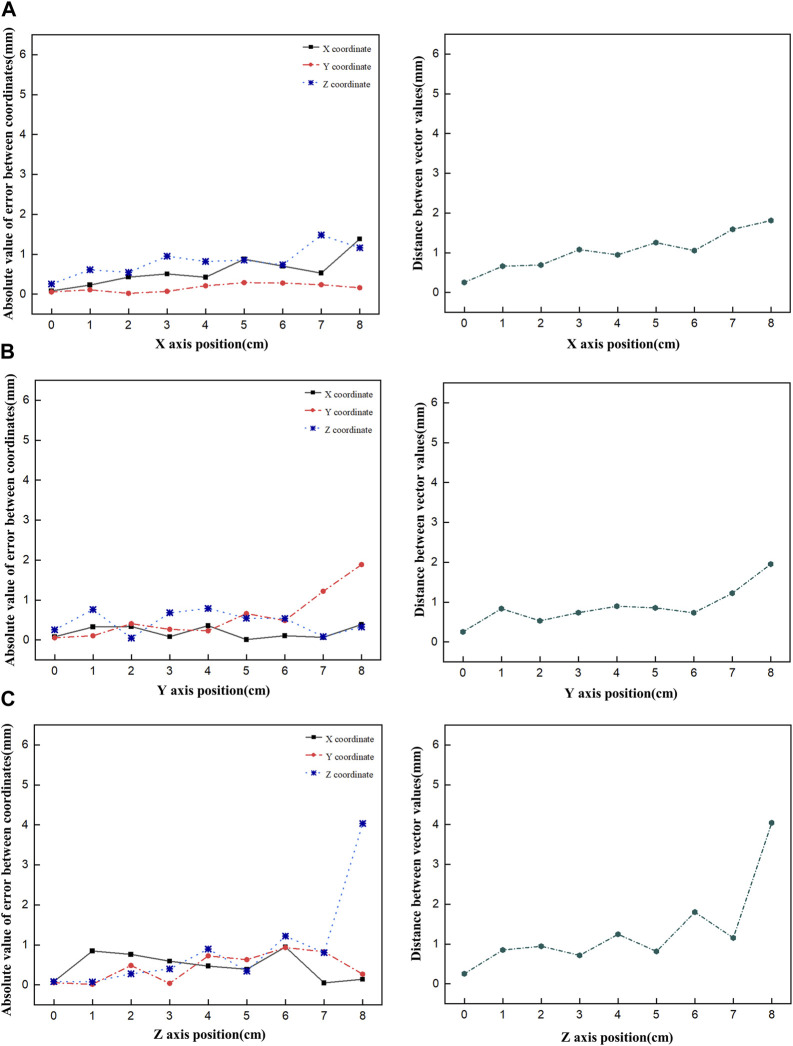
Plot of the absolute value of the error versus the Euclidean distance as the Ir-192 source is moved in each axis, moving the source in the *x*-axis **(A)**, *y*-axis **(B)**, and *z*-axis **(C)**, in that order.

First, the tracking system developed based on our proposed algorithm took 2.5–2.8 s to track the radiation source (Ir-192 source) and calculate its location. Figure 8 shows the absolute values of the x, y, and z errors in, and the Euclidean distance between, the 3D reconstructed coordinates derived from the acquired 2D images and the original source coordinates (as detailed in Tables 2–4). These data were recorded while systematically shifting the Ir-192 source along the x, y, or *z*-axis of the solid phantom. Figure 8A shows the absolute values of the errors in x, y, and z and the distance values recorded as the source was moved along the *x*-axis in 1-cm increments, while Figures 8B,C show the corresponding plots for movement of the source along the *y*-axis and *z*-axis directions, respectively. With the exception of the two largest source displacements (7 and 8 cm), the absolute values of the errors in the x, y, and z coordinates and the Euclidean distances are within 1 mm for almost every source position. When the source was moved 8 cm from the origin, the average absolute errors in the x-, y-, and z-coordinates were 0.440 mm, 0.423 mm, and 0.764 mm, respectively. Notably, displacement of the source along the *z*-axis by 8 cm led to a higher average error compared to the other axes. Furthermore, for most of the source displacements examined, the error in the z coordinate exceeded those in the x and y coordinates. Turning to the distance results, the overall average distance between the reconstructed and original positions across all source displacements was 1.146 mm. However, if 7 and 8 cm are excluded, the average distance is notably lower, at 0.889 mm.

**TABLE 2:** Original and reconstructed coordinates as the Ir-192 source was moved along the *x*-axis (mm).

Original coordinate of Ir-192	Reconstructed coordinate of Ir-192	Euclidean distance
(0, 0, 0)	(0.081, −0.054, 0.233)	0.253
(10, 0, 0)	(10.226, −0.109, 0.612)	0.661
(20, 0, 0)	(19.575, −0.018, 0.545)	0.691
(30, 0, 0)	(29.494, −0.069, 0.95)	1.079
(40, 0, 0)	(40.424, −0.209, 0.82)	0.947
(50, 0, 0)	(49.125, −0.288, 0.851)	1.254
(60, 0, 0)	(59.297, 0.276, 0.736)	1.055
(70, 0, 0)	(69.473, 0.234, 1.481)	1.589
(80, 0, 0)	(78.617, −0.16, 1.16)	1.812

**TABLE 3:** Original and reconstructed coordinates as the Ir-192 source was moved along the *y*-axis (mm).

Original coordinate of Ir-192	Reconstructed coordinate of Ir-192	Euclidean distance
(0, 0, 0)	(0.081, −0.054, 0.233)	0.253
(0, 10, 0)	(-0.326, 9.896, 0.761)	0.834
(0, 20, 0)	(0.335, 20.407, −0.047)	0.529
(0, 30, 0)	(0.082, 29.736, 0.681)	0.735
(0, 40, 0)	(0.358, 39.772, 0.790)	0.897
(0, 50, 0)	(0.010, 49.340, 0.544)	0.855
(0, 60, 0)	(-0.107, 59.515, 0.537)	0.731
(0, 70, 0)	(0.065, 68.782, −0.083)	1.223
(0, 80, 0)	(-0.388, 78.113, −0.326)	1.954

**TABLE 4:** Original and reconstructed coordinates as the Ir-192 source was moved along the *z*-axis (mm).

Original coordinate of Ir-192	Reconstructed coordinate of Ir-192	Euclidean distance
(0, 0, 0)	(0.081, −0.054, 0.233)	0.253
(0, 0, 10)	(-0.846, 0.013, 10.073)	0.849
(0, 0, 20)	(0.761, −0.484, 20.276)	0.943
(0, 0, 30)	(-0.592, −0.037, 30.397)	0.714
(0, 0, 40)	(0.468, 0.725, 40.887)	1.245
(0, 0, 50)	(-0.39, 0.629, 49.656)	0.816
(0, 0, 60)	(0.948, 0.932, 58.783)	1.802
(0, 0, 70)	(0.049, −0.827, 69.197)	1.154
(0, 0, 80)	(0.14, 0.267, 75.965)	4.046

**FIGURE 9:**
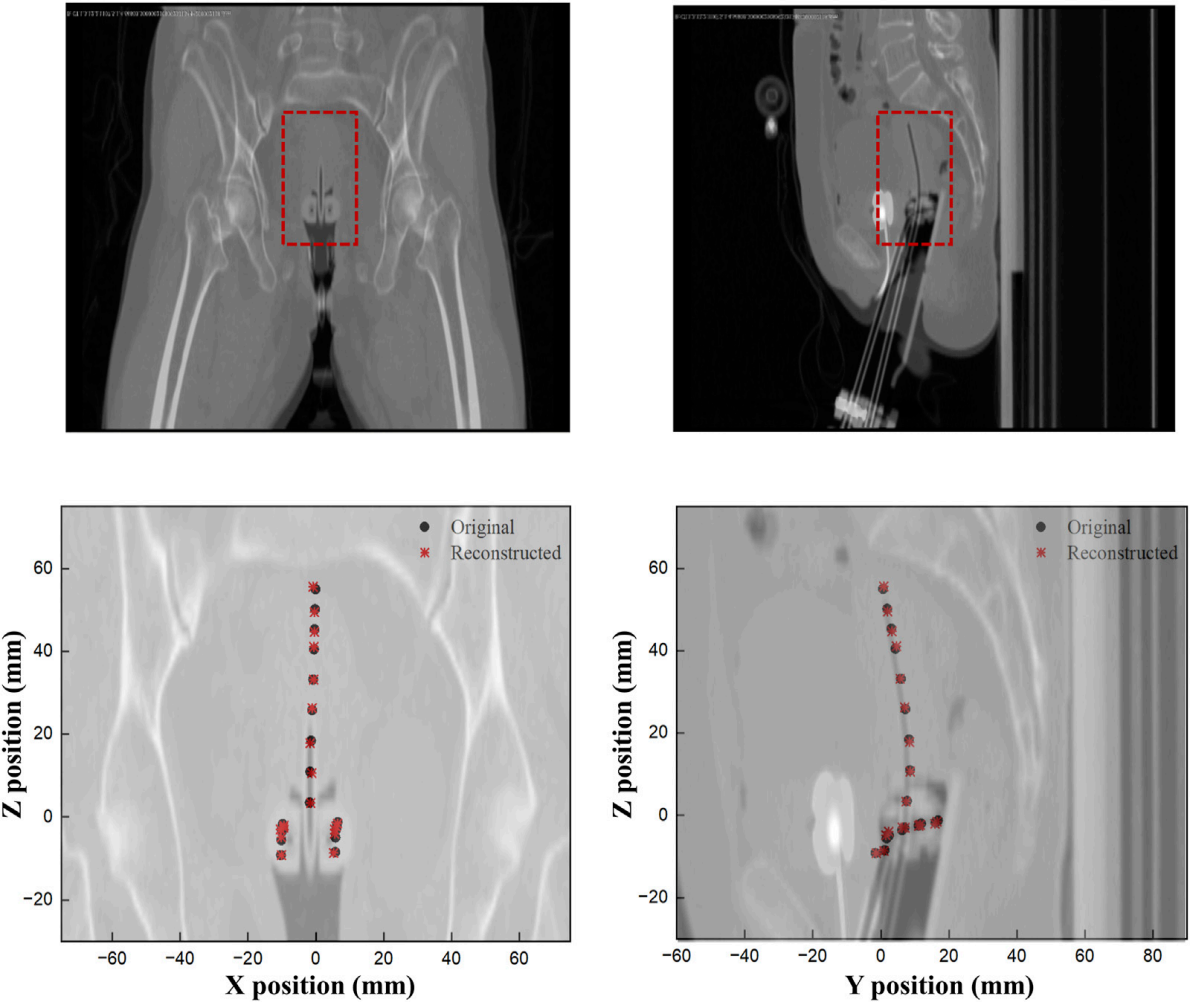
Comparison of the original and reconstructed source positions to show the difference in XY and ZY planes.

Finally, Figure 9 shows a comparison of the original and reconstructed positions of the Ir-192 source in the coronal and sagittal planes for 19 locations in a simulation based on data from a patient who underwent brachytherapy. The results show that the average errors in the x-, y-, and z-coordinates were 0.274, 0.178, and 0.401 mm, respectively, with ranges of 0.020–0.702, 0.012 to 0.327, and 0–0.909 mm, respectively (Table 5). Also, the mean distance between the original and reconstructed positions in the 19 cases was 0.586 mm (range, 0.251–0.957 mm).

**TABLE 5:** Original and reconstructed coordinates of patient data (mm).

Name of position	Original coordinate of Ir-192 source	Reconstructed coordinate of Ir-192 source	Euclidean distance
Ovoid1_1	(-9.8, 16, −1.7)	(-9.474, 15.841, −2.078)	0.524
Ovoid1_2	(-10, 11.1, −2.5)	(-9.554, 11.088, −2.558)	0.450
Ovoid1_3	(-10.1, 6.2, −3.6)	(-10.484, 6.289, −3.019)	0.702
Ovoid1_4	(-10.2, 1.7, −5.6)	(-10.234, 1.46, −4.897)	0.744
Ovoid1_5	(-10.3, −1.6, −9.2)	(-10.163, −1.351, −9.219)	0.285
Ovoid2_1	(6.455, 16.788, −1.249)	(6.348, 16.471, −1.593)	0.475
Ovoid2_2	(6.208, 11.851, −2.005)	(5.963, 11.524, −2.343)	0.530
Ovoid2_3	(5.975, 6.97, −3.044)	(5.54, 7.067, −3.044)	0.446
Ovoid2_4	(5.787, 2.366, −4.944)	(5.499, 2.275, −4.035)	0.958
Ovoid2_5	(5.719, 1.098, −8.473)	(5.218, 0.945, −8.705)	0.573
Tandem_1	(-0.129, 0.64, 55.0374)	(-0.831, 0.834, 55.586)	0.912
Tandem_2	(-0.264, 1.858, 50.19)	(-0.471, 1.951, 49.504)	0.723
Tandem_3	(-0.398, 3.077, 45.343)	(-0.378, 3.21, 44.704)	0.653
Tandem_4	(-0.533, 4.295, 40.495)	(-0.648, 4.573, 41.092)	0.669
Tandem_5	(-0.788, 5.882, 33.174)	(-0.673, 5.66, 33.2)	0.251
Tandem_6	(-1.094, 7.234, 25.803)	(-1.161, 6.988, 26.289)	0.549
Tandem_7	(-1.394, 8.298, 18.401)	(-1.697, 8.414, 17.817)	0.668
Tandem_8	(-1.675, 8.542, 10.911)	(-1.222, 8.633, 10.582)	0.567
Tandem_9	(-1.834, 7.703, 3.46)	(-1.511, 7.42, 3.307)	0.456

## 4 Discussion

HDR brachytherapy is capable of delivering a large radiation dose to a localized area in a short time, which leads to good patient prognosis. However, accurately determining the location of the radiation source during HDR brachytherapy treatment is made difficult by factors such as patient movement and machine malfunction. The resulting uncertainty in the location of the radiation source can lead to medical accidents (Poder et al., 2023). According to the safety standards for brachytherapy proposed by the American Association of Physicists in Medicine (AAPM) publications TG-43, 56, and 59, to minimize the above-mentioned uncertainties and prevent medical errors, operators are advised to perform QA tests to set the tolerance of the source position to ±1 mm and periodically verify that the source position is within the tolerance (Nath et al., 1995; Nath et al., 1997; Kubo et al., 1998; Rivard et al., 2004). Typically, however, the accuracy of the source location is not verified during a treatment session, and the position of the Ir-192 source in 3D, which varies over time, is also difficult to determine.

To solve these problems, operators need real-time 3D data on the movement of the source during treatment. The source positioning system also needs to be able to determine the position of the source during treatment, so patient safety and comfort are also important factors. Therefore, in the present study we developed an *in vivo* 4D tracking system based on multiple compact detectors capable of time-dependent stereoscopic 3D localization of radiation sources inside a patient’s body.

### 4.1 2D gamma images acquired using the optimized diverging collimator

Analysis of 2D gamma images acquired using simulations of the optimized diverging collimator revealed slightly distinct patterns in the FWHM and SNR values. The FWHM value remained within 1.1 mm as the source was moved from the center of the FOV along the *y*-axis direction or diagonal direction up to 6 cm, but increased markedly when the source was further displaced to 7 and 8 cm. The SNR value, by contrast, was sufficiently high (particularly, SNR values exceeding 25) up to a source displacement of 5 cm that the signals in the 2D images could be clearly distinguished. Nevertheless, when the source was moved beyond 6 cm, the noise increased noticeably, resulting in blurred signals. Based on these findings, we conclude that a detection system utilizing a diverging collimator possesses an effective range (in this study, ∼12 cm) within which movement of the Ir-192 source can be distinctly identified on the 2D image.

### 4.2 3D positioning using the proposed Ir-192 source tracking system

In our analysis of the accuracy of the tracking system, we found that the absolute value of the error in the reconstructed coordinates of the source in a solid phantom was within 1 mm for each axis, and that the Euclidean distances between the reconstructed and original coordinates were within 1.5 mm for all but two cases when the Ir-192 source was moved up to 6 cm along all axes. These results show a trend similar to that observed for the FWHM (spatial resolution performance) in the evaluation of 2D gamma images, but distinct from the trend observed for the SNR. This finding suggests that the 3D positioning accuracy of a multiple detector tracking system using the optimized diverging collimator is proportional to the spatial resolution performance of the 2D image detected through each individual detector. We also found that the range over which the system remains accurate in the 2D images is similar in 3D space for each of the x, y, and *z*-axes, which indicates that our proposed tracking system has an effective volume in 3D space (in this work, 12–13 cm for each axis).

Next, to ensure clinical usability, 3D positioning results based on patient data showed that the average distance between the reconstructed and original coordinates was 0.586 mm, which is a smaller error than when the source was moved along the x-, y- and *z*-axes using the solid phantom. This result can be interpreted as the experimental conditions for shift range of radiation source in the detector performance evaluation using the solid phantom, where the source’s range of motion extended up to a maximum of 8 cm from the center along the x, y, and *z*-axes. In contrast, the range of motion for 3D positioning data obtained from patient treatment data was observed to be within the mentioned effective range. The high accuracy of the proposed 4D tracking system in patient data demonstrates its clinical feasibility.

### 4.3 Comparison with previous research

Recently, other studies have been conducted to identify the 3d source position of the radiation source in HDR brachytherapy for more accurate and high-quality treatment. First, Yusuke Watanabe et al. developed an Ir-192 source tracking system utilizing a charge-coupled device (CCD) camera, dual pinhole collimator, and scintillator (Watanabe et al., 2018). In that study, to evaluate the accuracy performance of the tracking system, the source was placed in multiple locations within 2 ovoids and 1 tandem, like the evaluation method in our study, and the 3D distance between the original source position and the tracked source position was measured. When analyzing the numerical comparison between the two systems, it can be said that our system shows a better performance compared to the corresponding system in that the accuracy of the corresponding system shows an average value of 1.5 ± 0.7 mm, while the accuracy of our proposed tracking system shows 0.586 mm. However, considering that the results of this study are errors in a system that integrates hardware and software, it is somewhat difficult to say that we have conducted a comprehensive performance comparison between our proposed system and previously developed systems through numerical analysis only. In the future, if our research team also configures a hardware camera system compatible with the software system configured in this study to form an integrated tracking system, a more reasonable performance comparison between the two tracking systems will be possible.

Next, Roman Vasyltsiv et al. constructed and proposed a system for *in vivo* 4D tracking of an Ir-192 source utilizing a C-arm x-ray imager and tomosynthesis methods (Vasyltsiv et al., 2023). Their study was conducted using Monte Carlo simulations similar to ours and showed numerical accuracy ranging from 0 to 0.14 mm. Although the numerical accuracy of the system showed very good results, the limitation is that the source tracking requires continuous x-ray imaging to determine the location of the source, which causes radiation exposure to the patient. Both the system and our proposed system have their own advantages and disadvantages, but if we can improve the accuracy of our system through further research, our proposed 4D tracking system will be more competitive because it does not require additional radiation exposure.

### 4.4 Future work

Further research is needed to improve the 3D positioning accuracy of the proposed 4D tracking system composed of multiple compact detectors. First, efforts should be made to improve the effective range and volume of the system by varying the characteristics of the diverging collimator. In addition, systems with different numbers and placements of detectors should be studied to increase the detection accuracy when the source moves out of the effective volume. For example, our work on the current system suggests that it may be possible to minimize the performance degradation as the source moves away from the center by having sufficient cameras that when the source is positioned outside its effective range with respect to one camera, another camera compensates for the performance degradation. Additionally, in the proposed system the distance between the detector and the field of view (FOV) is almost constant, but it is also necessary to try to utilize 3D images with improved spatial resolution by adjusting the distance between the detector and the FOV.

Second, further research on collimators is needed. To date, diverging collimators and pinhole collimators have been used to acquire 2D gamma images when the size of the FOV is larger than the size of the actual detector. In this study, the detection system was configured using a diverging collimator, but future studies should test a similar detection system using an optimized pinhole collimator.

Finally, the current study used simulations to verify the feasibility of implementation. Future work in this area should seek to develop a hardware camera system that can detect the radiation source and to connect and integrate the system with a software system that performs positioning through 3D reconstruction for use in actual brachytherapy. In implementing the integrated solution, various detailed procedures such as acquiring gamma images through multiple cameras, transferring the acquired images to 3D positioning software, and performing 3D positioning will need to be considered, and detailed studies of each step will be required for more accurate and efficient source tracking.

## 5 Conclusion

In summary, this study proposed an *in vivo* 4D tracking system for HDR brachytherapy with an Ir-192 source that uses multiple compact detectors and 3D reconstruction. The proposed tracking system not only showed an error of <1 mm between the original and reconstructed positions of the source in 3D positioning using patient data, but also an average Euclidean distance between the two positions of <0.6 mm. These results indicate that the system has the potential to track the movement of Ir-192 sources in 3D space with high accuracy.

## Data Availability

The original contributions presented in the study are included in the article/Supplementary Material, further inquiries can be directed to the corresponding author.
